# Plasminogen activator inhibitor-1 is an independent prognostic factor of ovarian cancer and IMD-4482, a novel plasminogen activator inhibitor-1 inhibitor, inhibits ovarian cancer peritoneal dissemination

**DOI:** 10.18632/oncotarget.20834

**Published:** 2017-09-12

**Authors:** Erika Nakatsuka, Kenjiro Sawada, Koji Nakamura, Akihito Yoshimura, Yasuto Kinose, Michiko Kodama, Kae Hashimoto, Seiji Mabuchi, Hiroshi Makino, Eiichi Morii, Yoichi Yamaguchi, Takeshi Yanase, Akiko Itai, Ken-ichirou Morishige, Tadashi Kimura

**Affiliations:** ^1^ Department of Obstetrics and Gynecology, Osaka University Graduate School of Medicine, Suita, Osaka, Japan; ^2^ Department of Obstetrics and Gynecology, Gifu University Graduate School of Medicine, Gifu, Gifu, Japan; ^3^ Department of Pathology, Osaka University Graduate School of Medicine, Suita, Osaka, Japan; ^4^ IMMD Inc., Tokyo, Japan

**Keywords:** ovarian cancer, PAI-1, IMD-4482, peritoneal dissemination, angiogenesis

## Abstract

In the present study, the therapeutic potential of targeting plasminogen activator inhibitor-1 (PAI-1) in ovarian cancer was tested. Tissues samples from 154 cases of ovarian carcinoma were immunostained with anti-PAI-1 antibody, and the prognostic value was analyzed. Among the samples, 67% (104/154) showed strong PAI-1 expression; this was significantly associated with poor prognosis (progression-free survival: 20 vs. 31 months, P = 0.0033). In particular, among patients with stage II-IV serous adenocarcinoma, PAI-1 expression was an independent prognostic factor. The effect of a novel PAI-1 inhibitor, IMD-4482, on ovarian cancer cell lines was assessed and its therapeutic potential was examined using a xenograft mouse model of ovarian cancer. IMD-4482 inhibited *in vitro* cell adhesion to vitronectin in PAI-1-positive ovarian cancer cells, followed by the inhibition of extracellular signal-regulated kinase and focal adhesion kinase phosphorylation through dissociation of the PAI-urokinase receptor complex from integrin αVβ3. IMD-4482 caused G0/G1 cell arrest and inhibited the proliferation of PAI-1-positive ovarian cancer cells. In the xenograft model, IMD-4482 significantly inhibited peritoneal dissemination with the reduction of PAI-1 expression and the inhibition of focal adhesion kinase phosphorylation. Collectively, the functional inhibition of PAI-1 significantly inhibited ovarian cancer progression, and targeting PAI-1 may be a potential therapeutic strategy in ovarian cancer.

## INTRODUCTION

Ovarian cancer, which has a cure rate of only 30%, is generally characterized by widespread peritoneal dissemination and ascites and is the leading cause of death among gynecological cancers [1] and the 5-year survival rate remains at a dismal 30% and has not improved since the late 1990s [2]. Therefore, it is important to clarify the underlying pathological mechanism in the progression of ovarian cancer to generate improved therapeutic modalities.

Plasminogen activator inhibitor-1 (PAI-1/*SERPINE1*) is an endogenous inhibitor of urokinase-type plasminogen activator (uPA). PAI-1 normally functions as part of the plasminogen activation system, which includes the serine protease uPA, its receptor uPAR, and tissue-type plasminogen activator (tPA). Although the canonical view is that PAI-1 functions as an inhibitor of uPA and tPA to maintain clot formation, several reports have revealed that the urokinase plasminogen activator system, regulated by uPA, uPAR, and PAI-1, mediates proteolytic activity and the degradation of the basement membrane and extracellular matrix (ECM), which leads to tumor invasion and metastasis [3, 4]. In addition, it has been demonstrated that high levels of PAI-1 correlate with poor prognosis in various cancers including breast [5, 6], endometrial [7], gastric [8], colorectal [9], non-small cell lung [10], and renal cell cancer [11]. Regarding ovarian cancer, PAI-1 was found to be an independent prognostic factor in 86 patients with stage IIIc disease [12]. In contrast, van der Burg et al. failed to show a significant correlation between PAI-1 levels and prognosis in 90 patients with ovarian cancer, although PAI-1 expression was significantly increased in adenocarcinomas compared to normal tissue or benign adenomas [13]. Thus, comprehensive data regarding the prognostic value of the aberrant expression of PAI-1 in ovarian cancer remain limited, and its clinical impact among histologic subtypes remains unknown.

Despite many reports regarding PAI-1 expression in cancer cells, the role of PAI-1 in tumor progression remains controversial. For instance, while some authors have reported that PAI-1 inhibits cell adhesion and migration by blocking the binding of vitronectin (VN) to integrins or by dissociating bound uPAR from VN in the matrix [14, 15], others found that PAI-1 promotes cell adhesion of cancer cells [16, 17]. Thus, the role of PAI-1 is complicated, varying with both experimental design and the cellular origin of the protein. Therefore, more preclinical and mechanistic studies are required to elucidate the role of PAI-1 in human cancers.

With these factors in mind, we were encouraged to determine the prognostic value of PAI-1 expression in patients with ovarian cancer. Furthermore, we assessed the potential of a novel PAI-1 inhibitor, IMD-4482, as a therapeutic option in the management of ovarian cancer.

## RESULTS

### PAI-1 is an independent prognostic marker of ovarian cancer

**Figure 1:**
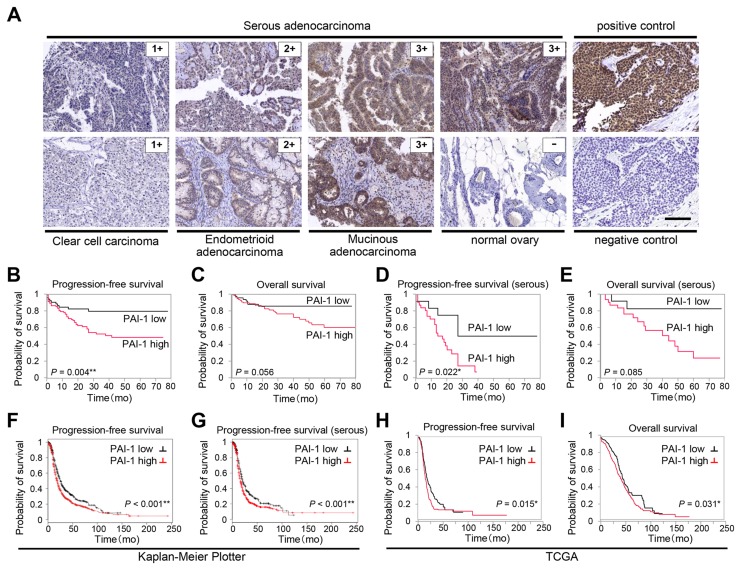
PAI-1 expression correlates with poor prognosis in patients with ovarian cancer Immunohistochemical staining of PAI-1 in different malignant ovarian tissue sections **(A).** Representative areas of three different ovarian cancers scored as 1, 2, and 3, and one normal ovarian tissue sample scored as 0. Sections from breast cancer are shown as positive control for PAI-1 staining, and as negative control stained with nonimmune sera. Bar, 100 μm. Kaplan-Meier plot showing progression-free survival (PFS) **(B)** and overall survival (OS) **(C)** of patients with ovarian cancer treated at Gifu University Hospital and Osaka University Hospital (n = 154) stratified by PAI-1 expression level. Kaplan-Meier plot showing PFS **(D)** and OS **(E)** of patients with serous adenocarcinoma (n = 54) stratified by PAI-1 expression level. Kaplan-Meier plot showing PFS of patients with ovarian cancer (n = 1307) **(F)** and PFS of patients with serous ovarian cancer (n = 1144) **(G)** stratified by PAI-1 gene expression. Patients are split by the threshold of lower tertile according to the microarray expression data for the probe representing *SERPINE*1 (202628_s_at). Kaplan-Meier plot showing PFS **(H)** (n = 395) and OS **(I)** (n = 485) of patients with stage II-IV high-grade serous ovarian cancer in TCGA database stratified by PAI-1 mRNA expression.

To elucidate the prognostic value of PAI-1 expression in ovarian cancer, ovarian cancer samples from 154 patients were collected and immunostained with anti-PAI-1 antibody. The characteristics of the patients are summarized in Table 1. The median age was 57 years, 54 cases (35.1%) were serous adenocarcinomas, and 37 cases (24.0%) were clear cell carcinoma (CCC). In Japan, the percentage of CCC is comparatively high, and the distribution of histologic types in the present study was comparable with that reported in Japan [18, 19]. Each sample was scored on the basis of the intensity of staining. Typically, clear cytoplasmic staining was seen in cases of positive PAI-1 expression (Figure 1A). Among the 154 tissue samples, 104 (67.5%) cases showed strong PAI-1 expression, 26 (16.9%) showed moderate expression, and 24 (15.6%) showed weak expression. Each PAI-1 staining score in all histological types was summarized in Supplementary Table 2. Thirty-eight of 54 (70.4%) in serous adenocarcinomas, 17 of 22 (77.3%) in endometrioid adenocarcinomas, 22 of 37 (59.5%) in clear cell carcinomas and 14 of 20 (70%) in mucinous adenocarcinomas showed strong PAI-1 expression. No significant differences were seen by histological types. None of the ovarian cancer tissue samples were scored as 0, while two out of three normal ovarian tissue samples showed negative PAI-1 expression, and one case showed weak expression. Among patients with ovarian cancer, those who had strong PAI-1 staining showed significantly worse progression-free survival (PFS) compared with those who had weak expression (median PFS: 19.5 vs. 28.5 months, P = 0.0041; Figure 1B). Strong PAI-1 staining also showed a trend toward worse overall survival (OS) compared to weak PAI-1 expression (median OS, 27.0 vs. 37.0 months, P = 0.0557; Figure 1C). Patients with serous histology were analyzed separately, as it is the most common subtype. Supplementary Table 1 shows the characteristics of patients with serous adenocarcinoma. Among 54 patients with stage II-IV serous adenocarcinoma, strong PAI-1 expression was an independent prognostic factor (median PFS: 13 vs. 19.5 months, P = 0.022; Figure 1D, median OS: 22 vs. 20.5 months, P = 0.085; Figure 1E). For multivariate analysis, a backward elimination approach was used to select a model for survival with multiple predictors. Age, International Federation of Gynecology and Obstetrics (FIGO) stage, histologic type, residual tumor at the time of surgery, and strong PAI-1 expression were entered in this model. The final model included strong PAI-1 expression as a significant independent predictor for reduced PFS in patients with ovarian cancer (hazard ratio [HR]: 2.45, 95% confidence interval [CI] 1.239–5.059, P = 0.005; Table 2).

**Table 1:** Clinicopathological characteristics of the patients

Number of patients	154
Median age, y (range)	57 (20-88)
Median observation time of patients alive, mo (range)	29 (1-145)
FIGO stage, n (%)	
I	69 (44.8)
II	13 (8.4)
III	50 (32.5)
IV	22 (14.3)
Histological feature, n (%)	
Serous papillary adenocarcinoma	54 (35.1)
Endometrioid adenocarcinoma	22 (14.3)
Clear cell carcinoma	37 (24.0)
Mucinous adenocarcinoma	20 (13.0)
Others	21 (13.6)
Residual tumor (cm), n (%)	
≤1	118 (76.6)
>1	36 (23.4)
Prior chemotherapy, n (%)	
Yes	11 (7.1)
No	143 (92.9)
Adjuvant chemotherapy, n (%)	
TC / ddTC	107 (69.5)
TC+THP	3 (1.9)
BEP	2 (1.3)
CPT-11+TXT	3 (1.9)
Others	5 (3.2)
None	34 (22.1)
Vital status, n (%)	
Living	120 (77.9)
Decreased	34 (22.1)
PAI-1 staining, n (%)	
Weak (1+)	24 (15.6)
Moderate (2+)	26 (16.9)
Strong (3+)	104 (67.5)

**Table 2:** Cox regression analysis of patients with ovarian cancer for progression free survival

	Univariate analysis			multivariate analysis		
	**HR**	**95% CI**	**p value**	**HR**	**95% CI**	**p value**
**Age (years)**						
< 50	1			1		
≥ 50	1.21	0.689-2.226	0.157	1.19	0.658-2.233	0.578
**FIGO Stage**						
I	1			1		
II-IV	32.9	10.20-201.1	*<0.001*	36.6	9.547-242.3	*<0.001*
**Histological type**						
serous	1			1		
endometrioid	0.18	0.044-0.515	*<0.001*	0.23	0.054-0.657	*0.004*
clear cell	0.39	0.173-0.790	*<0.001*	3.03	1.247-6.753	*0.018*
mucinous	0.14	0.022-0.463	*<0.001*	0.35	0.055-1.199	0.103
others	1.01	0.494-1.936	0.979	1.24	0.599-2.496	0.551
**Residual tumor (cm)**						
< 1	1			1		
≥ 1	6.41	3.710-11.12	*<0.001*	2.74	1.558-4.863	*<0.001*
**PAI-1 staining**						
low	1			1		
high	2.24	1.198-4.562	*0.010*	2.42	1.228-4.863	*0.005*

To strengthen our immunohistochemical data, a public access database of 1307 patients with ovarian cancer was analyzed (Kaplan-Meier plotter) [20, 21]. Patients with higher PAI-1 mRNA expression showed significantly shorter PFS compared with those with lower expression (Figure 1F). Among 1144 patients with serous adenocarcinoma, the patients with higher PAI-1 mRNA expression also showed significantly shorter PFS (Figure 1G). Among a sample of 603 patients with stage II-IV high-grade serous ovarian cancer obtained from The Cancer Genome Atlas (TCGA) database [22, 23], higher expression of PAI-1 mRNA was associated with significantly shorter PFS (median PFS: 13.0 vs. 15.4 months, P = 0.015, Figure 1H) as well as OS (median OS: 27.6 vs. 34.9 months, P = 0.031; Figure 1I).

### IMD-4482 inhibited PAI-1 activity in PAI-1-positive ovarian cancer cells

**Figure 2:**
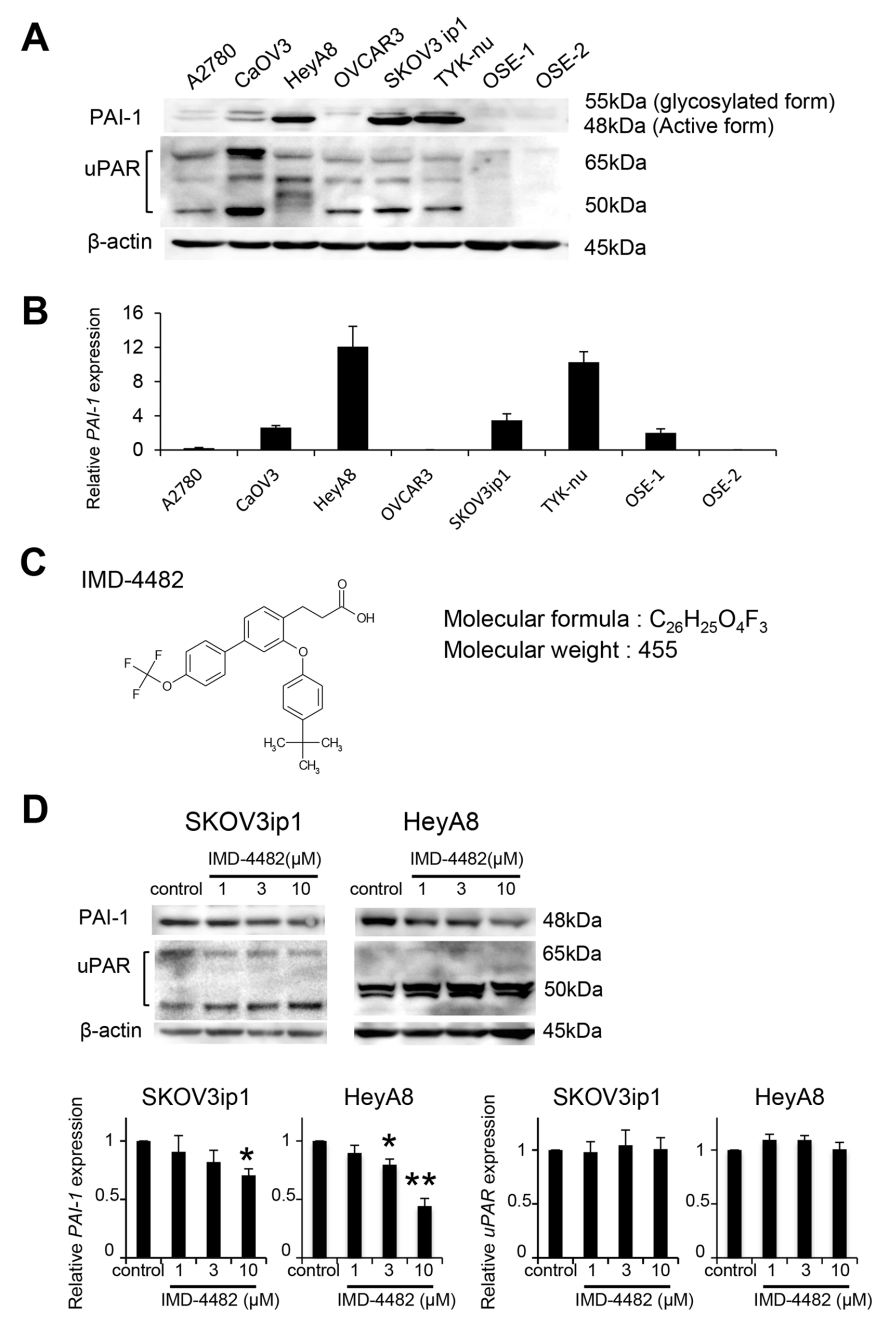
IMD-4482 inhibits PAI-1 activation of PAI-1-positive ovarian cancer cells PAI-1 expression in 6 serous ovarian cell lines and 2 different primary cultures of ovarian surface epithelium (OSE) cells was analyzed by western blot **(A)**. β-actin was used as a loading control. Real-time RT-PCR **(B)**. Total RNAs from the six cell lines and one OSE were collected and subjected to RT-PCR. The relative abundance of PAI-1 with respect to GAPDH expression was calculated. Molecular formula of IMD-4482 **(C)**. Western blot **(D)**. SKOV3ip1, HeyA8, and OVCAR3 cells were incubated with or without IMD-4482 in serum-free medium for 24 hours. Cell lysates were immunoblotted with antibodies against PAI-1 and uPAR. β-actin was used as a loading control. Experiments were repeated three times and are expressed as mean ± SD. ^*^; P < 0.05, ^**^; P < 0.01.

Because strong PAI-1 expression is significantly related to poor prognosis, particularly in serous adenocarcinoma, we decided to analyze the effect of PAI-1 inhibition on ovarian cancer cells. First, we assessed PAI-1 expression in six serous ovarian cancer cell lines. PAI-1 was robustly expressed in three out of six serous ovarian cancer cell lines (SKOV3ip1, TYK-nu, and HeyA8), while one cell line (OVCAR3) and two primary cultures of ovarian surface epithelial cells (OSE1 and 2) did not show detectable levels of PAI-1, and its receptor (uPAR) was expressed in all ovarian cancer cell lines tested (Figure 2A). Real-time RT-PCR confirmed similar trends in mRNA level (Figure 2B). IMD-4482, 3-[3-(4-tert-butylphenoxy)-4′- (trifluoromethoxy) biphenyl-4-yl] propanoic acid, a synthetic PAI-1 inhibitor, was supplied by IMMD Inc. (Tokyo, Japan) and used for the functional inhibition of PAI-1 in the subsequent experiments (Figure 2C). IMD-4482 inhibited the binding of tPA and PAI-1 (Supplementary Figure 1A). Additionally, in order to test whether the compound truly works as a PAI-1 inhibitor of uPA, the enzymatic activity of uPA was assessed by casein-plasminogen zymography in three ovarian cancer cell lines, SKOV3ip1 (PAI-1 positive), HeyA8 (PAI-1 positive) and OVCAR3 (PAI-1 negative) (Supplementary Figure 1B). Without treatment, only one gelatinolytic band of 48 kDa (high-molecular-weight uPA) was observed in all three cell lines. After treatment with 10 μM IMD-4482 for 24 hours, the PAI-1 positive ovarian cancer cell lines (SKOV3ip1 and HeyA8) displayed increased catalytic activity, as shown by a white band indicating 33 kDa (low-molecular-weight uPA), while the PAI-1-negative ovarian cancer cell line (OVCAR3) showed no detectable changes. Given that uPA is known to form a complex with PAI-1 and lose its catalytic activities, these zymography results suggest that IMD-4482 inhibited PAI-1 activities and increased uPA catalytic activities only in the PAI-1-positive cell lines. IMD-4482 treatment decreased PAI-1 expression in ovarian cancer cells in a dose-dependent manner (relative PAI-1 expression: SKOV3ip1: 1 μM, 0.91 ± 0.14, 3 μM, 0.82 ± 0.10, 10 μM, 0.71 ± 0.06; HeyA8: 1 μM, 0.90 ± 0.05, 3 μM, 0.81 ± 0.03, 10 μM, 0.45 ± 0.05), while it did not decrease uPAR expression (Figure 2D).

### IMD-4482 attenuated cell adhesion to vitronectin of PAI-1-positive ovarian cancer cells (SKOV3ip1 and HeyA8 cells), followed by inhibition of cell invasion

**Figure 3:**
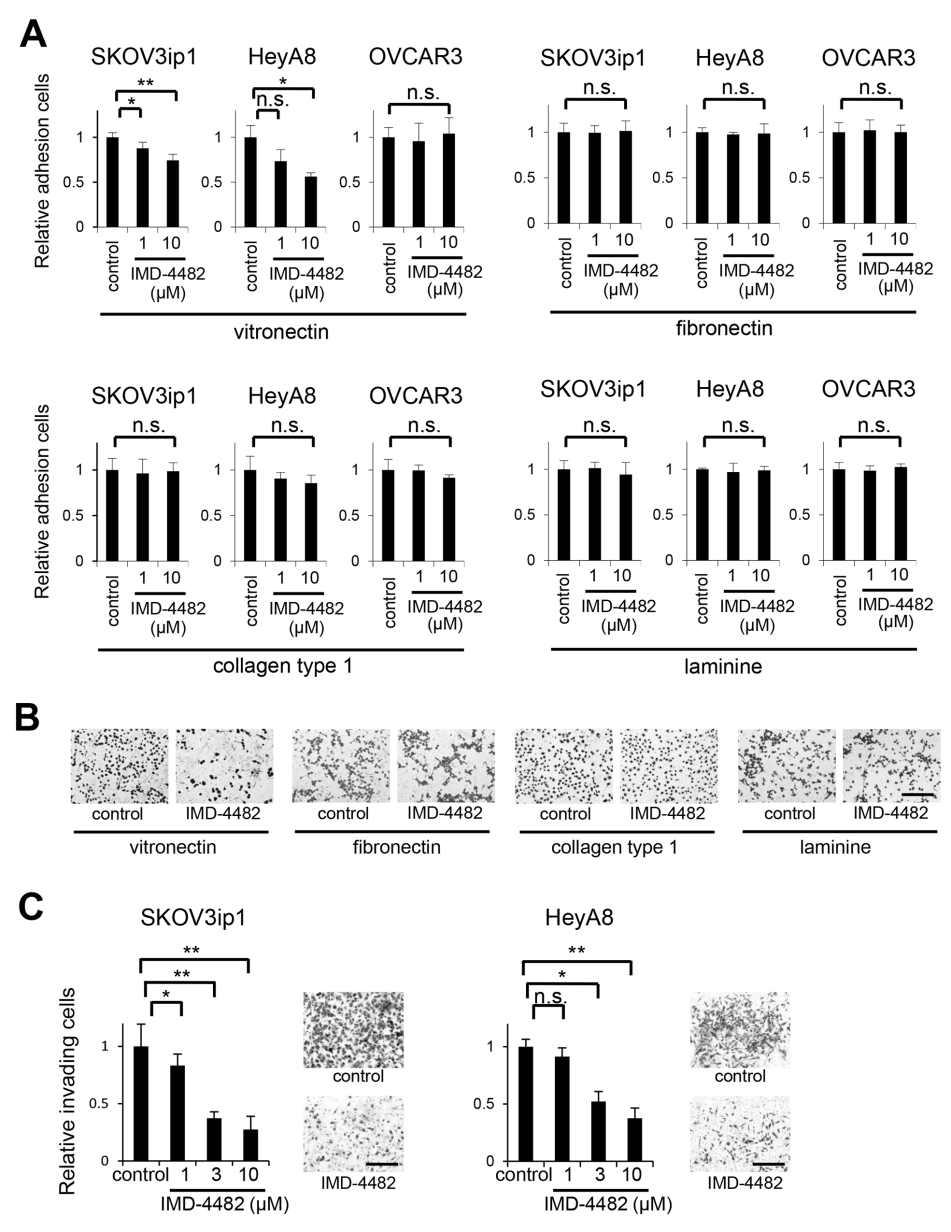
IMD-4482 inhibits adhesion and invasion of PAI-1-positive ovarian cancer cells (SKOV3ip1 and HeyA8 cells) but not PAI-1-negative cells (OVCAR3) *In vitro* adhesion assay **(A)**. A total of 1 × 10^5^ ovarian cancer cells (left, SKOV3ip1; middle, HeyA8; right, OVCAR3) were plated onto vitronectin-, fibronectin-, collagen type I-, and laminin-coated 96-well plates. After incubation for 50 minutes at 37°C, plates were washed to discard non-adherent cells, and the relative number of attached cells was measured, Data represents mean ± SD, n = 5 from triplicate independent experiments. **(B)** Representative images of *in vitro* adhesion assay of SKOV3ip1 cells (B). Bar, 200 μm. *In vitro* invasion assay **(C)**. A total of 4 × 10^4^ SKOV3ip1 (left) and 8 × 10^3^ HeyA8 (right) cells were plated on the top chamber in serum-free medium with IMD-4482, and allowed to invade for 48 hours. Invading cells on the underside of the filter were counted. Representative images are shown. Bar, 200 μm. Data represents mean ± SD, n = 5 from triplicate independent experiments. ^*^; P < 0.05, ^**^; P < 0.01, n.s.; not significant.

The functional role of PAI-1 in cell adhesion and migration remains controversial; while it has been recognized that PAI-I inhibits cell adhesion or migration by competing for VN binding sites with αVβ3 integrin and uPAR [14, 24], a recent study showed that the uPA–PAI-1 complex works as an agonist of uPAR-mediated cell adhesion on VN [17]. To investigate the effect of IMD-4482 on cell adhesion, we conducted *in vitro* cell adhesion assays onto different ECM ligands (fibronectin [FN], collagen type 1, VN, and laminin). IMD-4482 significantly inhibited the adhesion of PAI-1 positive ovarian cancer cells (SKOV3ip1, HeyA8) on VN (10 mmol/L: SKOV3ip1, 74%; HeyA8, 56%, respectively) in a dose-dependent manner, while the adhesion of OVCAR3 cells (PAI-1 negative) was not inhibited by IMD-4482 (Figure 3A and 3B). In order to further demonstrate that the involvement of PAI-1 in cell adhesion on VN, *SERPINE1* expression plasmid was transfected into OVCAR3 cells (Supplementary Figure 2A). MD-4482 significantly inhibited the adhesion of PAI-1 expressing OVCAR3 cells on VN (Supplementary Figure 2B). In contrast, the adhesion of these cells on other ECM ligands was not suppressed by IMD-4482, indicating that PAI-1 inhibition attenuated the cell adhesion specific to VN. Cell invasion follows adhesion. *In vitro* invasion assays revealed that IMD-4482 significantly impaired the invasion of PAI-1-positive ovarian cancer cells in a dose-dependent manner (3 mmol/L: SKOV3ip1, 37%; HeyA8, 52%; Figure 3C).

### IMD-4482 dissociated PAI-1 and uPAR from αV integrin and FAK, which led to inhibition of the phosphorylation of FAK and ERK

**Figure 4:**
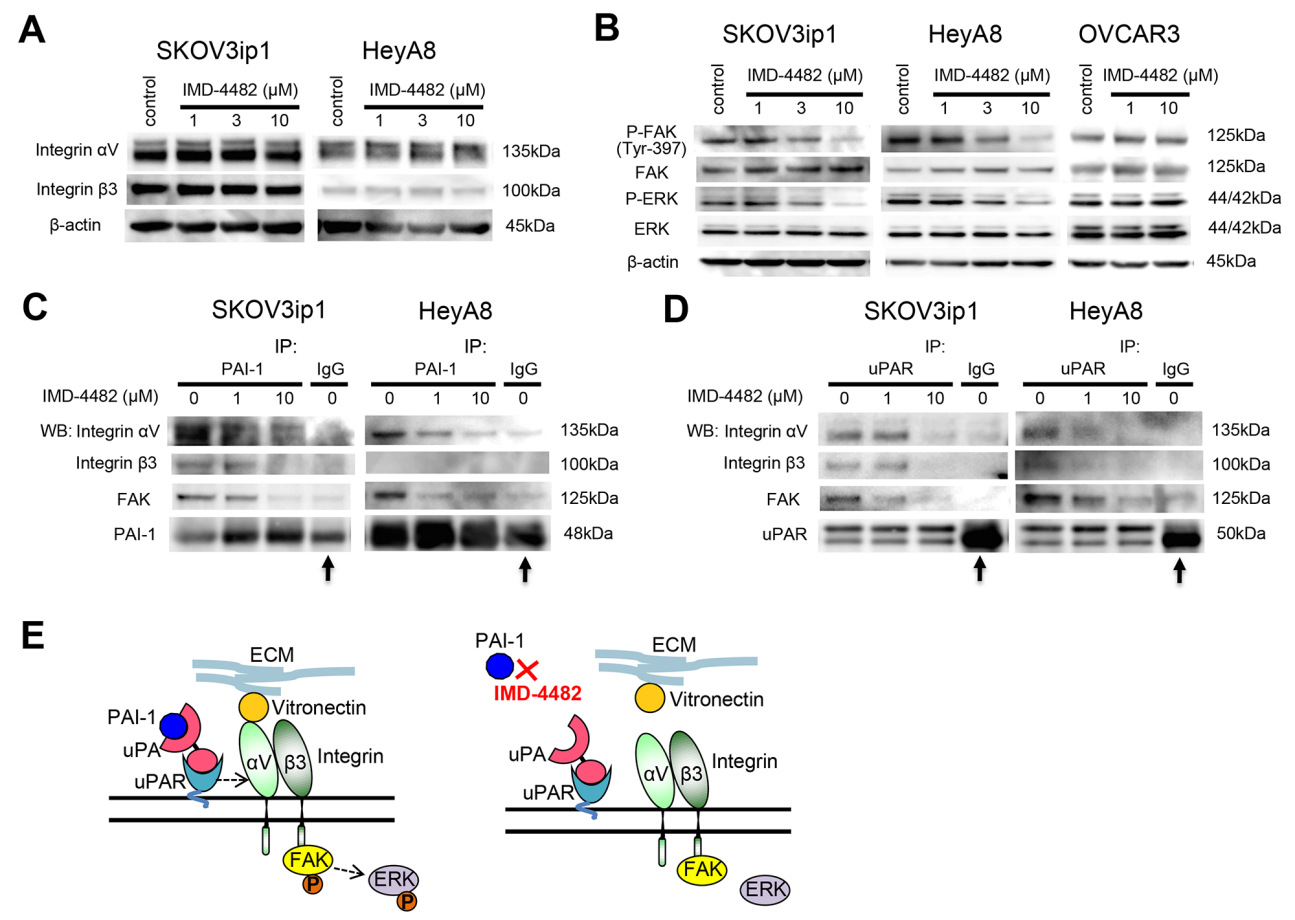
IMD-4482 inhibits the phosphorylation of FAK and ERK followed by the dissociation of PAI-1 and uPAR from αVβ3 integrin and FAK Western blot **(A)**. Cells were incubated with IMD-4482 for 24 hours. Cell lysates were immunoblotted with an antibody against integrin αV and integrin β3. β-actin was used as a loading control. Western blot **(B)**. Cells were incubated with or without IMD-4482 for 24 hours. Cell lysates were immunoblotted with an antibody against PARP, p-FAK (Tyr-397), FAK, p-ERK, and ERK. β-actin was used as a loading control. **(C** and **D)** Immunoprecipitation. Ovarian cancer cells were incubated with IMD-4482 for 24 hours, and lysed in buffer containing 1% Triton X-100. Cell lysates were subjected to immunoprecipitation with anti-PAI-1 antibody **(C)** or anti-uPAR antibody **(D)**, followed by immunoblotting to detect integrin αV, integrin β3, and FAK. Representative blots from three independent experiments are shown. Arrow indicates heavy chain of IgG band. Schema **(E)**. PAI-1 inhibition with IMD-4482 dissociates the interaction between PAI-1/uPAR and αVβ3 integrin, which leads to the inhibition of FAK phosphorylation.

Since VN is a major ligand for αVβ3 integrin, and this integrin-mediated adhesion is associated with the phosphorylation of focal adhesion kinase (FAK) [25, 26], the effect of PAI-1 inhibition on the expression of αVβ3 integrins as well as the phosphorylation of FAK and extracellular signal-regulated kinase (ERK), was examined. However, treatment with 10 μM IMD-4482 did not alter the expression of either αV integrin or β3 integrin (Figure 4A), while the drug successfully inhibited the phosphorylation of FAK and ERK in a dose-dependent manner in PAI-1-positive ovarian cancer cells (SKOV3ip1 and HeyA8 cells) but not OVCAR3 cells (Figure 4B). On the contrary, IMD-4482 inhibited the phosphorylation of FAK and ERK in PAI-1-exprresing OVCAR3 cells (Supplementary Figure 2C). Thus, we decided to analyze the manner in which PAI-1 inhibition affects the PAI-1/uPAR/αVβ3 integrin complex with the immunoprecipitation method. Immunoprecipitation using an anti-PAI-1 antibody revealed that treatment with IMD-4482 disrupted the interaction of PAI-1 with αVβ3 integrins and FAK (Figure 4C). This finding was further confirmed by immunoprecipitation with an anti-uPAR antibody showing that the interaction between uPAR and αVβ3 integrins and FAK was also disrupted by IMD-4482 (Figure 4D). These results indicate that IMD-4482 dissociated PAI-1 and uPAR from αVβ3 integrin and FAK, which led to inhibition of the phosphorylation of FAK and ERK (Figure 4E).

### IMD-4482 suppressed proliferation and induced G0/G1 cell cycle arrest followed by apoptosis

**Figure 5:**
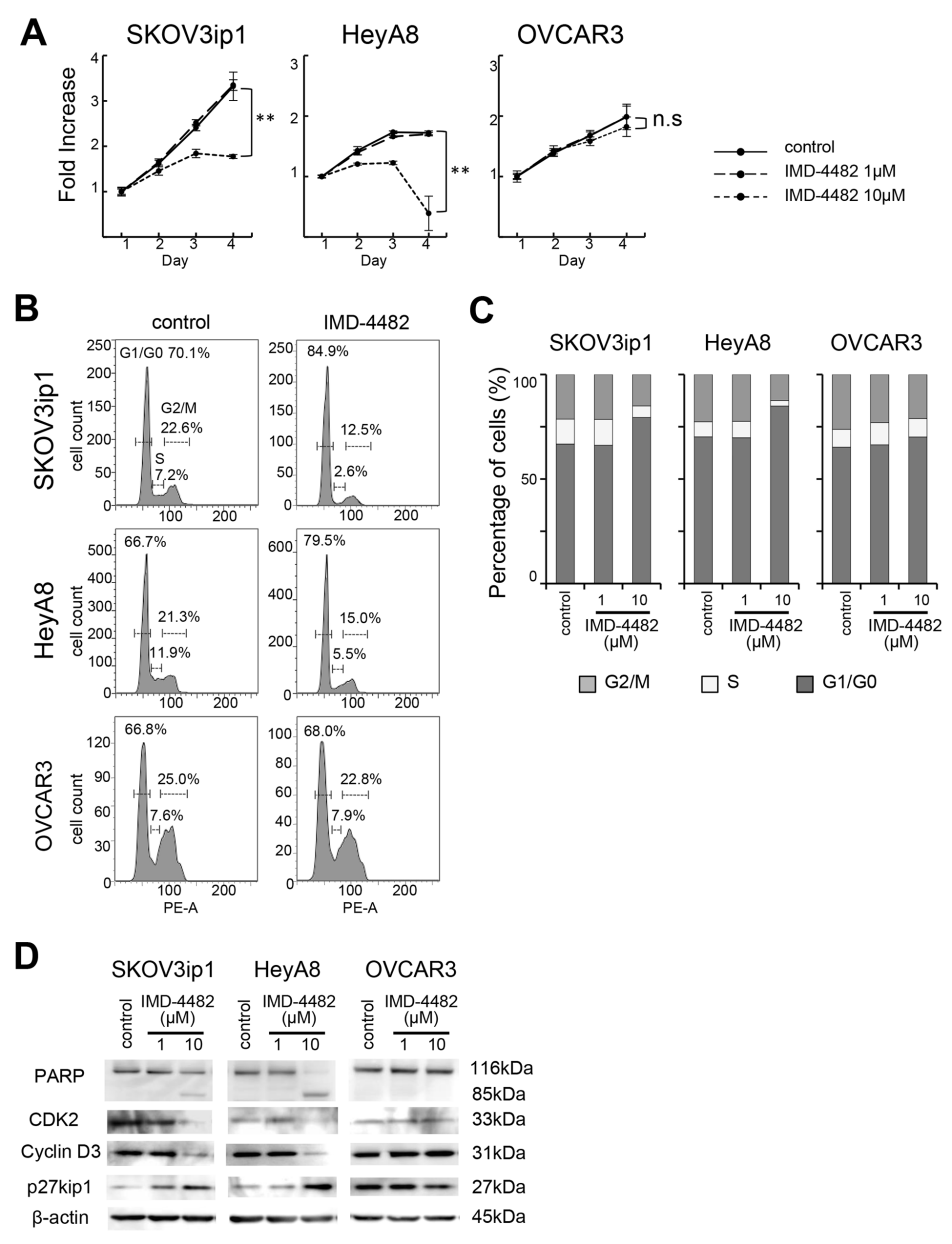
IMD-4482 suppressed proliferation and induced G0/G1 cell cycle arrest and apoptosis in PAI-1-positive ovarian cancer cells Effect of IMD-4482 on cell proliferation **(A)**. SKOV3ip1, HeyA8, and OVCAR3 cells were plated onto 96-well plates and cultured in DMEM containing 2% FBS with or without IMD-4482. Effect of IMD-4482 on cell cycle distribution **(B and C)**. Cells treated with or without IMD-4482 for 24 hours were stained with propidium iodine and analyzed by flow cytometry. Representative flow histograms (B), and percentages of cells in G0/G1, S, and G2/M phase (C) are shown. Western blot **(D)**. Cells were incubated with or without IMD-4482 for 24 hours. Cell lysates were immunoblotted with antibodies against PARP, CDK2, cyclin D3, and p27kip1. β-actin was used as a loading control.

Since the phosphorylation of ERK is known to be associated with cell proliferation and viability, we next evaluated the effects of IMD-4482 on cell proliferation. We found that 10 μM of IMD-4482 significantly inhibited the proliferation of PAI-1-positive cells (SKOV3ip1, HeyA8), but had no effect on PAI-1-negative cells (OVCAR3) (Figure 5A), while IMD-4482 significantly inhibited the proliferation of PAI-1 expressing OVCAR3 cells (Supplementary Figure 2D). Cell cycle analysis with fluorescence-activated cell sorting (FACS) revealed that IMD-4482 significantly increased the proportion of cells in G0/G1-phase and decreased the proportion of cells in S-phase among PAI-1-positive cells (SKOV3ip1, HeyA8), while this effect was not observed in PAI-1-negative cells (OVCAR3) (Figures 5B and C), indicating that PAI-1 inhibition with IMD-4482 caused G0/G1 arrest. Thus, expression of the cell cycle-related proteins associated with G0/G1-phase was analyzed (Figure 5D). IMD-4482 induced the cleavage of poly (ADP-ribose) polymerase (PARP) in PAI-1-positive ovarian cancer cells (SKOV3ip1 and HeyA8) but not in PAI-1-negative cells (OVCAR3), indicating that the drug induced apoptosis through PAI-1 inhibition. IMD-4482 inhibited the depletion of the G1-phase transition complexes, cyclin D3 and CDK2, in parallel with upregulation of the cell cycle inhibitor p27Kip1 in SKOV3ip1 and HeyA8 but not OVCAR3 cells. Collectively, IMD-4482 reduced cellular proliferation through an inability to progress from the G0/G1 phase of the cell cycle, which leads to apoptosis in PAI-1-positive ovarian cancer cells.

### IMD-4482 suppressed proliferation and induced apoptosis following G0/G1 cell cycle arrest in paclitaxel-resistant ovarian cancer cell lines

Because ovarian cancer eventually acquires resistance to chemotherapy including taxanes and platinum and this is the major obstacle to improved prognosis, new molecular-targeted therapies should be clinically employed mainly in chemoresistant cases. For this reason, we were interested in the efficacy of PAI-1 inhibition in paclitaxel-resistant ovarian cancer cells. First, paclitaxel-resistant sublines from SKOV3ip1 cells and HeyA8 cells were developed in our laboratory by continuous exposure to paclitaxel (Supplementary Figure 3A), and these were named SKOV3ip1/T and HeyA8/T cells, respectively. Both SKOV3ip1/T and HeyA8/T cells expressed PAI-1 as strongly as did their parental cells (Supplementary Figure 3B). Thereafter, the effect of PAI-1 inhibition with IMD-4482 on proliferation, apoptosis, and the phosphorylation of FAK and ERK was analyzed. It was found that 10 μM of IMD-4482 significantly inhibited the proliferation of these resistant cells (Supplementary Figure 3C), induced the cleavage of PARP, and inhibited the phosphorylation of FAK and ERK (Supplementary Figure 3D), indicating that PAI-1 inhibition may be effective in chemoresistant cases. Thus, IMD-4482 has the potential to be employed in the clinical setting.

### IMD-4482 inhibits peritoneal dissemination of ovarian cancer cells through the inhibition of FAK phosphorylation and the attenuation of intratumoral vessel formation

**Figure 6:**
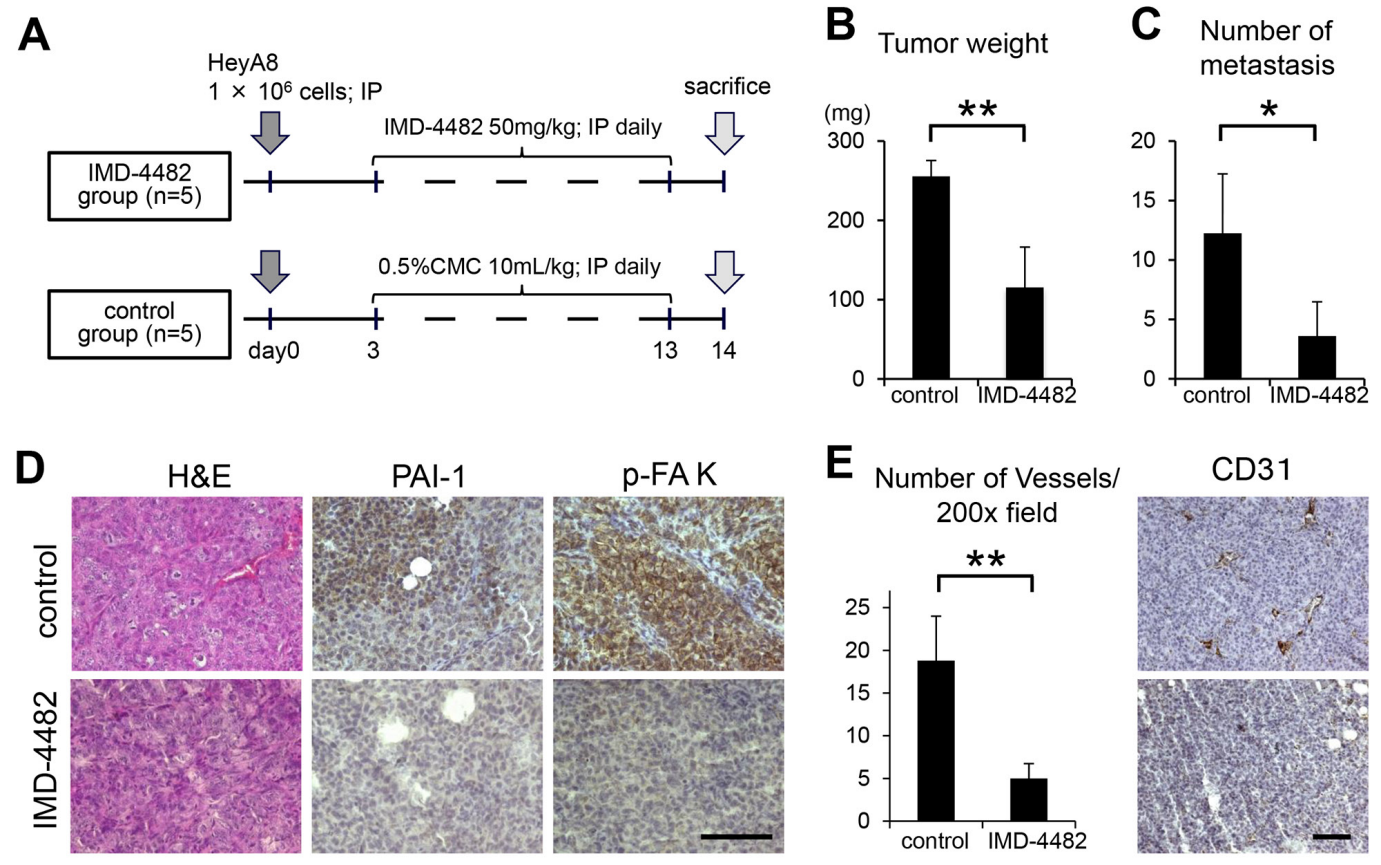
IMD-4482 inhibits peritoneal dissemination of ovarian cancer cells through inhibition of FAK phosphorylation and intratumoral vessel formation Experimental protocol **(A)**. A total of 1 × 10^6^ HeyA8 cells were injected intraperitoneally into female BALB/c nu/nu mice. Three days after the injection, IMD-4482 (50 mg/kg body weight) or an equal amount of 0.5% CMC-Na (control) was injected intraperitoneally daily for 11 days. Effect of IMD-4482 on intraperitoneal tumor weight **(B)** and number of metastases **(C)**. Results are expressed as mean ± SD, each n = 5. Representative tumor areas were stained with H&E, and immunostained with antibodies against PAI-1 and p-FAK **(D)**. Bar, 50 mm. Number of microvessels per field by CD31 staining (×200) **(E,** right). Results are expressed as mean ± SD, n = 5, each. The representative tumor areas immunostained with an antibody against mouse CD31 are shown **(E,** left). Bar, 50 μm. ^*^, P < 0.05; ^**^, P < 0.01; n.s., not significant.

Finally, the therapeutic potential of IMD-4482 in a xenograft mouse model of ovarian cancer was examined. HeyA8 (1 × 10^6^ cells) were injected intraperitoneally into female BALB/c nu/nu mice. Three days after the inoculation, mice showed multiple tumor dissemination to the omentum, the peritoneal surface, the surface of the liver, and the small bowel mesentery. Thus, treatment was initiated on that day (Figure 6A). Eleven days after the treatment, both the tumor weight and the number of peritoneal implants were significantly inhibited in mice treated with IMD-4482 compared with controls (0.5% carboxymethylcellulose [CMC]) (tumor weight: IMD-4482; 115.4 ± 51.0 vs. control; 255.5 ± 20.1 mg, P < 0.01, number of peritoneal implants: 3.6 ± 2.9 vs. 12.3 ± 5.0, P < 0.05, respectively; Figure 6B and 6C). To analyze the mechanism of action of IMD-4482 *in vivo*, immunohistochemical analyses were performed on samples from inoculated tumors (Figure 6D). The expression of PAI-1 as well as the phosphorylation of FAK was inhibited in tumors treated with IMD-4482 compared with controls, suggesting that IMD-4482 inhibited the progression of ovarian cancer by inhibiting the phosphorylation of FAK in cancer cells.

Several reports have revealed that PAI-1 induces angiogenesis. For instance, Isogai et al. reported that PAI-1 promotes angiogenesis by stimulating endothelial cell migration toward FN [27]. Thus, in order to assess the effect of IMD-4482 on angiogenesis, the intratumoral vessels as revealed by anti-mouse CD31 staining were analyzed. Treatment with IMD-4482 significantly inhibited the number of intratumoral vessels (5.0 ± 1.7 vs. 18.8 ± 5.2/field [×200], respectively, P < 0.01; Figure 6E).

## DISCUSSION

A significant association between high expression of PAI-1 in tumors and poor prognosis in various types of tumors has been confirmed [5–11]. While conflicting results had been reported regarding ovarian cancer, we have shown, in the present study, that strong PAI-1 expression are seen in approximately two-thirds of ovarian cancer samples and PAI-1 expression is an independent prognostic factor among patients with stage II-IV serous adenocarcinoma, suggesting that targeting PAI-1 in ovarian cancer therapy remains an attractive approach particularly in serous adenocarcinoma, the most common and aggressive subtype. Our immunohistochemical data were further confirmed using large data sets provided by Kaplan-Meier plotter and TCGA. Mashiko et al. reported similar results using a microarray data set from the Australian Ovarian Cancer Study (AOCS) [28]. Recently, van Dam et al. performed a systematic Med-line search and showed overexpression of u-PA and PAI-1 can be found in more the 75% of primary ovarian cancer samples as well as in most metastatic samples [29]

In the present study, IMD-4482 inhibited *in vitro* cell adhesion to VN in PAI-1-positive ovarian cancer cells, followed by the inhibition of ERK and FAK phosphorylation through the dissociation of the PAI-1/uPA/uPAR complex from integrin αVβ3. PAI-1 has been reported to have dual roles in cell adhesion. Since uPA functions as an anti-adhesion molecule through proteolytic degradation of ECM components, PAI-1 would be expected to promote adhesion [30]. In contrast, PAI-1 also exhibits an anti-adhesive function. PAI-I inhibits uPAR-mediated cell adhesion and migration by interacting with VN [14, 15]. Most recently, De Lorenzi et al. [17] demonstrated that complex formation between PAI-1 and uPA enhances cell adhesion to VN by at least three different mechanisms: First, the reaction of PAI-1 with uPA attenuates plasminogen activation and therefore the proteolytic inactivation of VN. Second, the PAI-1/uPA complex works as a strong and irreversible agonist of uPAR binding to VN. Third, complex formation with uPA results in the release of PAI-1 from VN, liberating binding sites for adhesion receptors. Our *in vitro* data strongly support these findings.

In the present study, we revealed that PAI-1 inhibition attenuated cell proliferation and induced apoptosis following G0/G1 arrest in PAI-1-positive ovarian cancer cells. This result is supported by several previous studies that examined the effect of PAI-1 on apoptosis, cell proliferation, and cell signaling. Increased expression of PAI-1 is reported to be associated with resistance to apoptosis in the human promyelocytic leukemia cell line HL-60 [31]. PAI-1 promotes cell proliferation of MCF-7 cells in the presence of uPA by sustaining ERK phosphorylation [32], and promotes the proliferation of vascular smooth muscle cells through the activation of NF-κB and ERK [33]. Thus, inhibition of sustained ERK phosphorylation with IMD-4482 is likely to be associated with the attenuation of cell proliferation. Regarding the cell cycle, Giacoia et al. [34] reported that downregulation of PAI-1 significantly reduced cellular proliferation owing to an inability to progress from the G0/G1 phase of the cell cycle in human urothelial cell lines and HeLa cell lines, findings similar to those reported in the present study.

Over the last two decades, several laboratories and pharmaceutical companies have developed a variety of small-molecule PAI-1 inhibitors [35]. The focus of investigations on the activity of these inhibitors had been in cardiovascular diseases (thrombus repermeabilization), lung fibrosis, or Alzheimer's disease, and to a lesser degree in cancer [35]. According to the recent review by Placencio et al. [36], several small-molecule PAI-1 inhibitors have been tested in preclinical models of cancer. When administered to not only PAI-1-positive Lewis lung carcinoma but also to PAI-1-negative B16 melanoma tumor-bearing mice, SK-216, a specific PAI-1 inhibitor, caused a significant reduction in subcutaneous primary tumor size and inhibited angiogenesis and metastases, suggesting the involvement of host PAI-1 during tumor progression [37]. The oral administration of PAI-039, a small molecule PAI-1 inhibitor, to mice xenotransplanted with human T24 bladder and HeLa cervical cancer cells resulted in a significant reduction of tumor volume with a decrease in tumor cell proliferation and vascularization and an increase in apoptosis [38]. Regarding ovarian cancer, Mashiko et al. recently reported that TM5275, another small molecule PAI-1 inhibitor, effectively blocked *in vitro* cell proliferation of ovarian cancer cells that highly expressed PAI-1 [28]. In the present study, for the first time, we demonstrated that IMD-4482 significantly reduced uPAR/αVβ3 integrin-mediated adhesion to VN, followed by the reduction of FAK and ERK phosphorylation and inhibition of cell proliferation as a result of G0/G1 arrest, leading to a novel rationale that inhibition of PAI-1 can be a potential therapy for ovarian cancer.

In a xenograft study, we showed that IMD-4482 significantly inhibited intratumoral vessel formation, indicating that this drug has the potential to work as an antiangiogenic drug. However, to date, PAI-1 has been reported to exert both proangiogenic and antiangiogenic effects. The proangiogenic effect of PAI-1 is mediated through its antiprotease function, leading to stabilization of the ECM [39], or by stimulating endothelial cell migration toward FN [27]. In contrast, PAI-1 is also reported to block αVβ3 integrin binding to VN, and hence, inhibit angiogenesis [14]. More recently, Wu et al. showed that PAI-1 inhibits angiogenic signaling by uncoupling vascular endothelial growth factor receptor (VEGFR2)-2/αVβ3 integrin cross talk [40]. Masuda et al. commented that the systemic administration of SK-216, a PAI-1 specific inhibitor, reduced tumor angiogenesis, raising the possibility that systemic administration of a PAI-1 inhibitor could become a novel antiangiogenic therapy in the treatment of malignancies [37]. The precise mechanism of PAI-1 involvement in tumor angiogenesis has not been fully understood, and further investigation is required.

Overall, the mechanism underlying PAI-1 control of tumor progression is so complex and pleiotropic that it appears to be incompletely understood at present. The present study might provide a step toward understanding the role of PAI-1 in mediating cell adhesion and cell signaling, indicating that a small-molecule PAI-1 inhibitor such as IMD-4482 could become a potential therapeutic agent for ovarian cancer, given that approximately two thirds of ovarian cancers show high PAI-1 expression associated with poor prognosis.

## MATERIALS AND METHODS

### Materials

IMD-4482, a synthetic PAI-1 inhibitor, was supplied by IMMD Inc. (Tokyo, Japan). Growth factor-reduced basement membrane proteins (Matrigel), human FN, collagen type 1, VN, laminin, integrins Sampler Kit (#611435; antibodies against integrins αV and β3), and an antibody against FAK (#610087) were purchased from BD Biosciences (Franklin Lakes, NJ, USA). Antibodies against PAI-1 (H-135), VEGF (A-20), CD31 (M-20), and normal mouse IgG (sc-2027) were purchased from Santa Cruz Biotechnology (Dallas, TX, USA). Antibodies against uPAR (#9692), PARP (#9542), CDK2 (78B2), Cyclin D3 (DCS22), p27 kip1 (D69C12), Phospho-p44/42 MAPK (p-Erk 1/2: Thr 202/Tyr 204) (E10), p44/42 MAPK (Erk 1/2) (3A7), and β-actin (#4967) were obtained from Cell Signaling Technology (Danvers, MA, USA). Antibody against p-FAK (Tyr-397) (44-624G) was purchased from Life Technologies (Carlsbad, CA, USA). Human *SERPINE1* expression plasmid (HG10296-UT) and control vector (pCMV) were purchased from Sino Biological Inc. (Beijing, China).

### Cell culture

The ovarian cancer cell line, SKOV3ip1 cells (serous adenocarcinoma, mutation; PIK3CA, ARID1A, amplification; ERBB2; [41]) were kindly provided by Dr. Ernst Lengyel (University of Chicago, Chicago, IL, USA) in 2007. HeyA8 cells (serous adenocarcinoma, mutation; KRAS, BRAF) were kindly provided by Dr. Anil Sood (MD Anderson Cancer Center, Houston, TX, USA). A2780 (serous adenocarcinoma, mutation; PIK3CA, PTEN, BRAF, ARID1A) and CaOV3 (serous adenocarcinoma, mutation; TP53) were purchased from American Type Culture Collection (ATCC; Rockville, MD, USA) in 2007. OVCAR3 (serous adenocarcinoma, mutation; TP53, C11orf30, CCNE1) was provided by the Cell Resource Center for Biomedical Research Institute of Development, Aging and Cancer of Tohoku University (Sendai, Japan). TYK-nu (serous adenocarcinoma, mutation; TP53) was purchased from the Japanese Collection of Research Bioresources Cell Bank (Osaka, Japan). Cells were cultured in DMEM supplemented with 10% FBS, 100 U/mL penicillin, and 100 μg/mL streptomycin and incubated in 95% air/5% CO_2_ at 37°C. Cells were authenticated by short tandem repeat DNA profiling at Takara-Bio Inc. (Kusatsu, Japan) and were used for this study within six months of resuscitation.

Primary cultures of human ovarian surface epithelium (OSE) cells were established according to the method of Auersperg et al. [42]. Briefly, normal ovarian tissues were obtained from women undergoing oophorectomy for benign disease, and the ovarian epithelia were scraped from the underlying ovarian cortex and transported in Hanks’ buffered salt solution (Gibco, Paisley, UK) before centrifugation. OSE cells were then resuspended in 199-MCDB medium (Sigma-Aldrich, St. Louis, MO, USA) with 15% FBS medium and antibiotics (100 U/mL penicillin, 100 μg/mL streptomycin), and cultured in this medium in a 95% air/5% CO2 atmosphere at 37°C.

### Tissue collection and immunohistochemistry

A total of 154 ovarian carcinoma samples were collected; 76 samples at Osaka University Hospital (Osaka, Japan) between 2007 and 2013, and 78 samples at Gifu University Hospital (Gifu, Japan) between 2006 and 2011. Three normal ovarian tissue samples were included as a control. All samples were sliced and stained with hematoxylin and eosin (H&E), and confirmed to contain viable tumorous areas plus nontumorous areas.

Tissue sections were deparaffinized in xylene and rehydrated with 100% ethanol. For antigen unmasking, tissue sections were put in a Pascal pressure chamber (Dako, Carpinteria, CA, USA) in Target Retrieval Solution (pH 9.0; Nichirei Biosciences, Tokyo, Japan). After undergoing blocking with blocking solution (Dako), sections were incubated with the primary PAI-1 antibody at 1:50 for 1 hour at room temperature and washed with TBS-T. They were then stained using N-Histofine Simple Stain MAX PO(R) (Nichirei Biosciences) for 30 minutes, and DAB chromogen (Dako) was added as a chromogen staining substrate for 5 minutes. Sections were then counterstained with hematoxylin, dehydrated, and coverslipped. For negative controls, another section was incubated with mouse immunoglobulin (Dako) instead of primary antibody. Slides were intensively examined by two independent qualified pathologists (EM and SM) without knowledge of the clinical outcomes. Each sample was scored on the basis of the intensity of the staining (0, no staining; 1, weak; 2, moderate; 3, strong). “High” expression of PAI-1 was defined as a score of 3, and “low” expression was defined as a score of 0 to 2.

### Kaplan-Meier plotter analysis

The prognostic value of the PAI-1 genes in ovarian cancer was analyzed using Kaplan-Meier plotter [20, 21], a database that integrates gene expression data and clinical data. To date, Kaplan-Meier plotter is capable of assessing the effect of 54,675 genes on survival using 10,188 cancer samples, including samples from 1,648 ovarian cancer patients. Patients were split by the threshold of lower tertile according to the microarray expression data for the probe representing *SERPINE1* (202628_s_at), and the two patient groups (higher and lower PAI-1 gene expression levels) were compared using a Kaplan-Meier survival plot.

### TCGA analysis

The prognostic value of the PAI-1 mRNA in ovarian cancer was analyzed using the TCGA database [22, 23], which includes mRNA expression data from 603 patients with stage II-IV high-grade serous ovarian cancer. We downloaded the PAI-1 (*SERPINE1*) mRNA expression data and clinical information for these patients. Patients were split into two groups (higher and lower PAI-1 mRNA expression levels) by the PAI-1 mRNA expression Z score threshold of -0.4, and the two groups were compared using the Kaplan-Meier method.

### Western blot analysis

A total of 5 × 10^5^ cells were plated onto 6-well plates and lysed with 1 × RIPA Buffer (25 mM Tris-Hcl pH 7.6, 150 mM NaCl, 1% NP-40, 0.1% SDS, 1% sodium deoxycholate, 1 mM PMSF, protease inhibitor cocktail; 1:100 [Nacalai Tesque, Inc., Kyoto, Japan]). Lysates (15 μg) were separated by 10% or 5–20% gradient sodium dodecyl sulfate-polyacrylamide gel electrophoresis (SDS-PAGE; Wako Pure Chemical Industries, Ltd., Osaka, Japan), and transferred to polyvinylidene difluoride membranes, followed by incubation with the primary antibodies (PAI-1, 1:200; uPAR 1:1000; PARP, 1:1,000; CDK2, 1:500; Cyclin D3, 1:2,000; p27 kip1, 1:500; p-ERK 1/2, 1:1,000; ERK1/2, 1:1,000; p-FAK, 1:1,000; FAK 1:1,000; Integrin αV, 1:250; Integrin β3, 1:2500; β-actin, 1:2,000) in TBS-T containing 20% Bullet Blocking One (Nacalai Tesque) and then with a corresponding secondary horseradish peroxidase-conjugated IgG. The proteins were visualized with an electrochemiluminescent system (PerkinElmer, Waltham, MA, USA).

### qRT-qPCR analysis

Total RNA was extracted using TRIzol Reagent (Life Technologies) according to the manufacturer's instructions. cDNA synthesis was performed with 800 ng of total RNA using a ReverTra Ace® qPCR RT Master Mix (FSQ-201) (Toyobo, Osaka, Japan). RT-qPCR reactions were performed using the StepOnePlus Real-Time PCR System (Applied Biosystems, Foster City, CA, USA) with the following probes from Applied Biosystems: PAI-1 (Hs01126606_m1), and human glyceraldehyde-3-phosphate dehydrogen (GAPDH: 4326317E) as an internal control. Comparative real-time PCR was performed in triplicate, and relative levels of PAI-1 expression were calculated using the 2-ΔΔCt method.

### *In vitro* cell proliferation assay

SKOV3ip1 (1 × 10^3^ cells/well), HeyA8 (1.2 × 10^3^ cells/well), and OVCAR3 (2 × 10^3^ cells/well) cells were seeded onto 96-well plates and cultured in DMEM supplemented with 2% FBS with increasing concentrations of IMD-4482 ranging from 1.0 to 10 μM. IMD-4482 was prepared as a 100 mmol/L stock solution in dimethyl sulfoxide (DMSO) and DMSO alone was employed as a control. After the time points indicated in the figure, cells were washed three times with serum-free medium and frozen at -80°C until analysis. Cell proliferation was measured using the CyQUANT® Cell Proliferation Assay Kit (Life Technologies, #C7026) according to the manufacturer's instructions.

### Matrigel invasion assay

*In vitro* cellular invasion was assayed by determining the ability of cells to invade through a synthetic basement membrane. Briefly, 8-μm pore size polycarbonate filters coated with 25 μg Matrigel (BD Biosciences) were placed in a modified Boyden chamber. Ovarian cancer cells at a density of 4 × 10^4^ cells/well were plated onto the top chamber in serum-free medium and incubated with medium containing 2% FBS as a chemoattractant in the bottom chamber. After the cells were allowed to invade through the Matrigel barrier for 48 hours, the filter was fixed and stained with Giemsa solution. Non-invading cells were removed using a cotton swab and invading cells on the underside of the filter were counted using an inverted microscope.

### *In vitro* adhesion assay to ECM components

Ovarian cancer cells at a density of 1 × 10^5^ cells/well were plated in a 96-well plate precoated with 50 μg/mL FN, 50 μg/mL collagen type I, and 50 μg/mL laminin or 2 μg/mL VN. The concentration of ECMs was determined based on previous studies [43, 44]. After incubation for 50 min at 37°C, cells were washed two times with PBS to discard non-adherent cells, fixed with methanol, and stained with Giemsa solution. The number of adhesive cells was quantified by adding 100 μL of 0.2% Triton X to lyse cells and measure the absorbance at 560 nm with a microplate absorbance reader (SH9000Lab; Hitachi, Tokyo, Japan).

### Cell cycle analysis

A total of 1 × 10^6^ cells were seeded onto 6-cm dishes. After pretreatment with IMD-4482 at concentrations ranging from 1.0 to 10 μM in DMEM for 24 hours, cells were harvested by brief trypsinization, washed, and fixed with 75% ethanol at -20°C overnight. Cells were then incubated with 100 μg/mL of RNase (Sigma-Aldrich) at 37°C for 20 minutes, and stained with 50 μg/mL of propidium iodide (Sigma-Aldrich). After incubation for 1 hour in the dark at room temperature, cells were analyzed by flow cytometry on a FACScan™ cytometer (BD Biosciences, Bockville, MD, USA) using FlowJo version 7.6.5 software (Tree Star Inc., Ashland, OR, USA).

### Immunoprecipitation

A total of 1 × 10^6^ cells were seeded onto 6-cm dishes and then starved in serum-free medium with IMD-4482 or the equivalent volume of DMSO for 24 hours. The cells were washed three times with ice-cold PBS and lysed with immunoprecipitation buffer (150 mM NaCl, 50 mM Tris-HCl pH 7.6, 1% Triton X-100, 1 mM PMSF, and protease inhibitor cocktail; 1:100 [Nacalai Tesque]). A sample of 200 μg (200 μL) of cell extract was subjected to pre-clearing with protein A beads (Adar Biotech, Rehovot, ISRAEL), and the beads were removed by centrifugation at 14,000 rpm for 10 minutes. Pre-cleared lysates were incubated with 1 μg of PAI-1 antibodies (H-135) (Santa Cruz Biotechnology) or 3 μL of uPAR antibodies (D4Q5S) (Cell Signaling Technology) overnight at 4°C with rotation. As a negative control, 3uL of normal rabbit IgG (sc-2027) (Santa Cruz Biotechnology) was used instead of the primary antibody. A volume of 40 μL of 50% Protein A bead slurry was then added, and a further 2-hour incubation at room temperature was performed. The immune complexes were pelleted by centrifugation at 1,600 rpm for 3 minutes, washed three times with immunoprecipitation buffer without protease inhibitors, and then subjected to immunoblotting as described above.

### Animal experiments

All animal experiments were approved by the Institutional Animal Care and Use Committee of Osaka University (Osaka, Japan), in accordance with institutional and National Institutes of Health (NIH) guidelines. Female athymic BALB/c nude mice (aged 5 weeks) were purchased from CLEA Japan Inc. (Tokyo, Japan). A total of 1 × 10^6^ HeyA8 cells were suspended as single cells in a volume of 0.2 mL PBS containing 0.1% BSA and injected intraperitoneally into the mice. Mice were assessed daily for general health. Three days after the inoculation, the mice were intraperitoneally administered either 0.5 mL of 0.5% carboxymethylcellulose sodium salt (CMC-Na) or IMD-4482 (50 mg/kg) every day for 11 days and finally sacrificed. The number of metastases in each mouse was counted, the metastases were carefully dissected, and the removed tumors were weighed. Tumor tissues were immediately fixed in formalin, embedded in paraffin, and sliced into 5-μM sections with a microtome. The slides were prepared and incubated with PAI-1 antibody at 1:50, p-FAK antibody at 1:200, and mouse CD-31 at 1:300 for 1 hour at room temperature, and stained as described above. Five representative images of tumors were taken randomly, and the number of intratumoral vessels as visualized with anti-mouse CD31 per field (×200) was counted.

### Statistical analysis

JMP Pro version 11.2.0 (SAS Institute Japan Ltd., Tokyo, Japan) was used for statistical analyses. Data were expressed as means ± standard deviation (SD). Differences were analyzed using the Mann-Whitney *U* test. Survival estimates were calculated using the Kaplan-Meier method, and comparisons between groups were analyzed using the log-rank test. Univariate analysis and multivariable models were fit using a Cox proportional hazards regression model. A backward elimination approach was used to fit the multivariable model so that variables achieving significance at the α = 0.05 level would be included. Differences were considered statistically significant at P < 0.05.

## SUPPLEMENTARY MATERIALS FIGURES AND TABLES


