# Supplementary material for: Plasminogen activator inhibitor-1 is an independent prognostic factor of ovarian cancer and IMD-4482, a novel plasminogen activator inhibitor-1 inhibitor, inhibits ovarian cancer peritoneal dissemination
Source: Oncotarget. 2017 Sep 12;8(52):89887–902. doi: 10.18632/oncotarget.20834 (PMC5685717; doi:10.18632/oncotarget.20834)
Supplement: Supplementary file 1 [file oncotarget-08-89887-s001.pdf]

Plasminogen activator inhibitor-1 is an independent prognostic factor of ovarian cancer and IMD-4482, a novel plasminogen activator inhibitor-1 inhibitor, inhibits ovarian cancer peritoneal dissemination

SUPPLEMENTARY MATERIALS

tPA Binding assay

75 nM of recombinant PAI-1 and different dose of IMD-4482 were mixed and incubated in PBS at 37°C for 5 min, and then 150 nM of tPA was added. After incubation in PBS at 37°C for 5 min, samples were applied for 10% SDS-PAGE and visualized with a silver staining. The bands of tPA/PAI-1 complex were quantified by a densitometry.

Plasminogen zymography

Plasminogen zymography was performed as previously described [1] [2]. Briefly, 90-100% confluent cells were cultured in serum free DMEM with increasing concentrations of IMD-4482 for 24 h, and conditioned medium were collected. Samples were centrifuged at 4000 rpm for 10 min at 4°C, and protein concentrations were measured using the Bradford method-based Bio-Rad assay. Protein extracts (30 µg) were separated on 10% SDS-PAGE containing 0.2 mg/ml casein and 20 µg/ml human plasminogen (Wako Pure Chemical Industries, Ltd., Osaka, Japan). Gels were washed twice for 30 min in 2.5% Triton X-100 to remove the SDS and then incubated overnight in the incubation buffer [50 mM Tris-HCl (pH 7.6), 0.15 M NaCl, 10 mM $CaCl_2$, 0.05% NaN_3] overnight at 37°C. Finally, gels were stained with Coomassie blue and then destained until contrast was satisfactory.

Establishment of chemoresistant cell lines

Two paclitaxel-resistant cell lines, namely SKOV3ip1/T and HeyA8/T cells were developed in our laboratory by continuously exposing their parental cells to paclitaxel. Cells were initially exposed to a paclitaxel concentration of 1 nM, and after the cells had regained their exponential growth rate, the concentration of paclitaxel was increased gradually, and the procedure was repeated until the concentration was 300 nM.

MTS assay

Cell viabilities were determined by MTS assay with CellTiter 96 AQueous One Solution Reagent (Promega, Madison, WI, USA) according to the manufacturer's instruction. Cells were plated in 96-well plates, and exposed to paclitaxel at increasing concentrations for 48 hours before the assays were performed. After the addition of MTS for 1 hour, the number of surviving cells was assessed by measure the absorbance at 490 nm. Each experiments were repeated at least three times.

REFERENCES

1. Heussen C, Dowdle EB. Electrophoretic analysis of plasminogen activators in polyacrylamide gels containing sodium dodecyl sulfate and copolymerized substrates. Anal Biochem. 1980; 102:196–202.
2. Adhami F, Yu D, Yin W, Schloemer A, Burns KA, Liao G, Degen JL, Chen J, Kuan CY. Deleterious effects of plasminogen activators in neonatal cerebral hypoxia-ischemia. Am J Pathol. 2008; 172:1704–16.

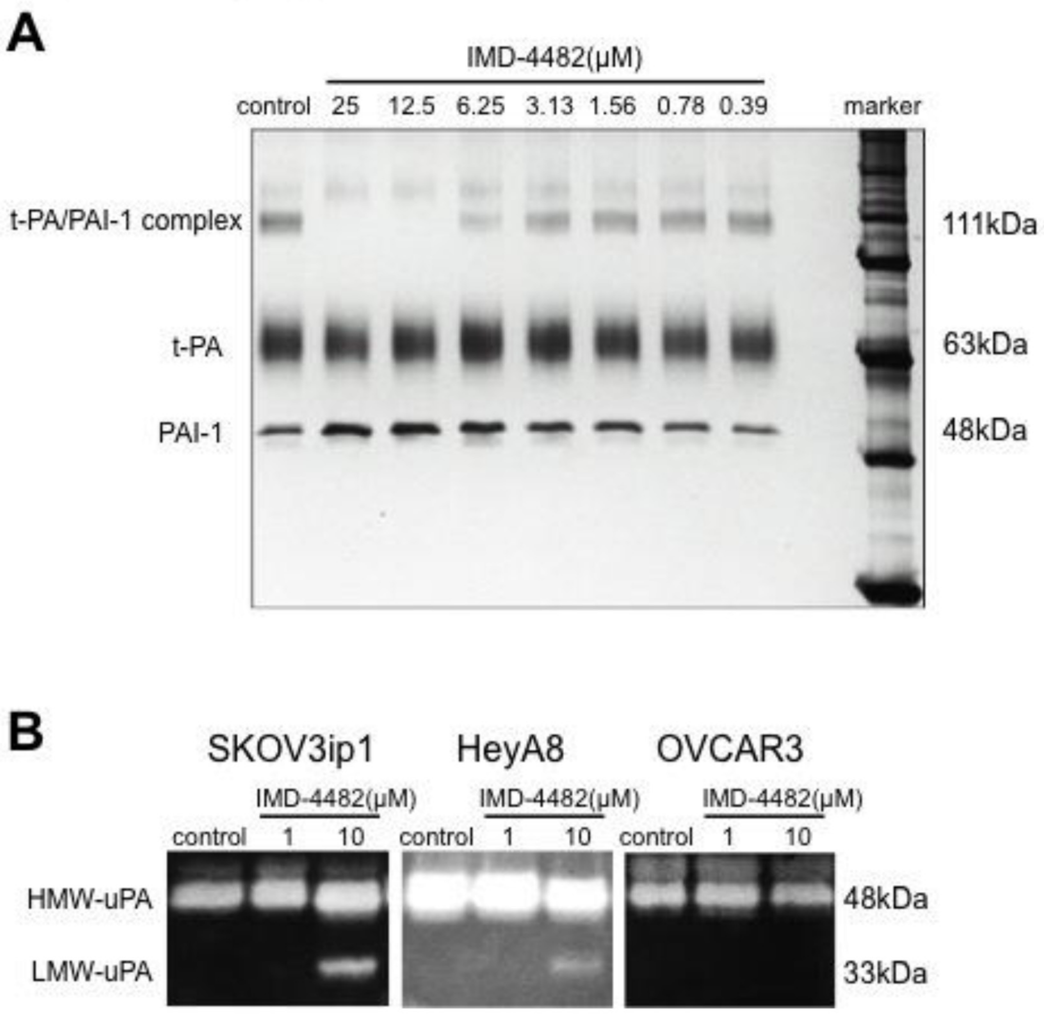

Supplementary Figure 1: The effect of IMD-4482 on tPA and uPA activity. Inhibitory effects of IMD-4482 on the binding of tPA and PAI-1 **(A)**. The recombinant PAI-1 and IMD-4482 were mixed, and then tPA was added. After incubation at 37°C for 5 min, samples were applied for 10% SDS-PAGE and visualized with a silver staining. Plasminogen zymography **(B)**. Cells were treated with or without IMD-4482 for 24 hours, and supernatants were resolved by electrophoresis on plasminogen plus casein gel. The gels were rinsed with 2.5% Triton X-100 and were stained with 0.25% coomassie blue.

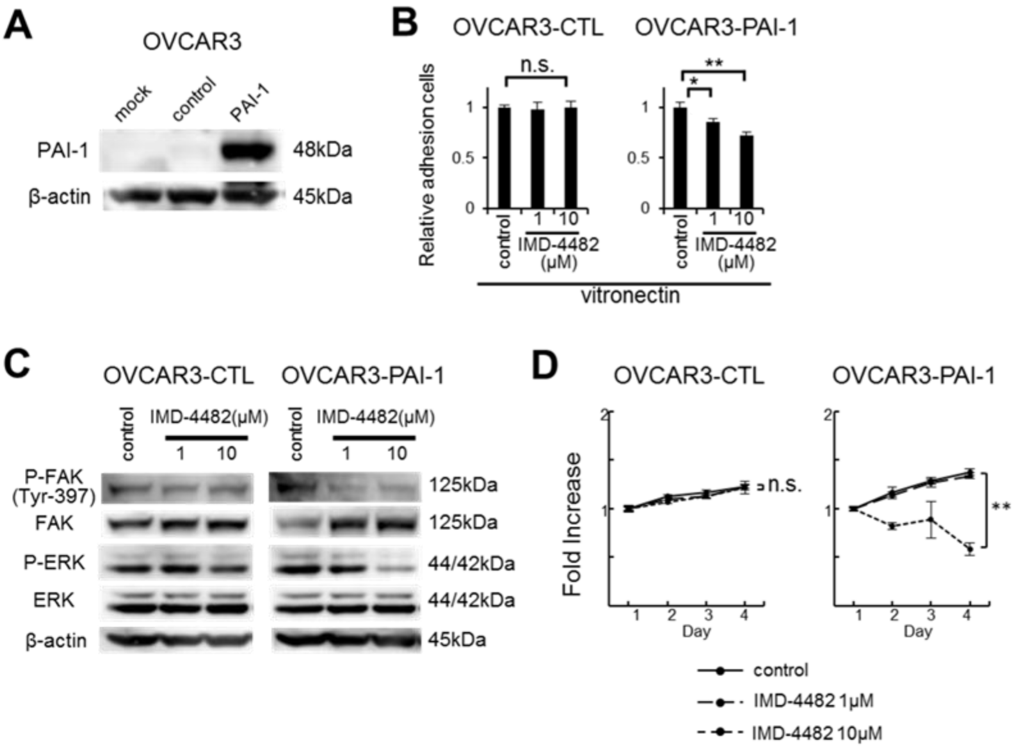

Supplementary Figure 2: Western Blot (A). Control vector (control) or PAI-1 expression vector (PAI-1) was tranfected into OVCAR-3 cells. Cell lysates were immunoblotted with an antibody against PAI-1. β-actin was used as a loading control. *In vitro* adhesion assay **(B)**. A total of 1 × 10^5 OVCAR-3 cells transfected with control vector (OVCAR3-CTL) or PAI-1 expression vector (OVCAR3-PAI-1) were plated onto vitronectin-coated 96-well plates. After incubation for 50 minutes, plates were washed to discard non-adherent cells, and the relative number of attached cells was measured, Data represents mean ± SD, n = 5 from triplicate independent experiments. Western blot **(C)**. Cells were incubated with or without IMD-4482 for 24 hours. Cell lysates were immunoblotted with an antibody against PARP, p-FAK (Tyr-397), FAK, p-ERK, and ERK. β-actin was used as a loading control. In vitro cell proliferation assay **(D)**. OVCAR3 cells transfected with control vector (OVCAR3-CTL) or PAI-1 expression vector (OVCAR3-PAI-1) were plated onto 96-well plates and cultured in DMEM containing 2% FBS with or without IMD-4482. **, P < 0.01; n.s., not significant.

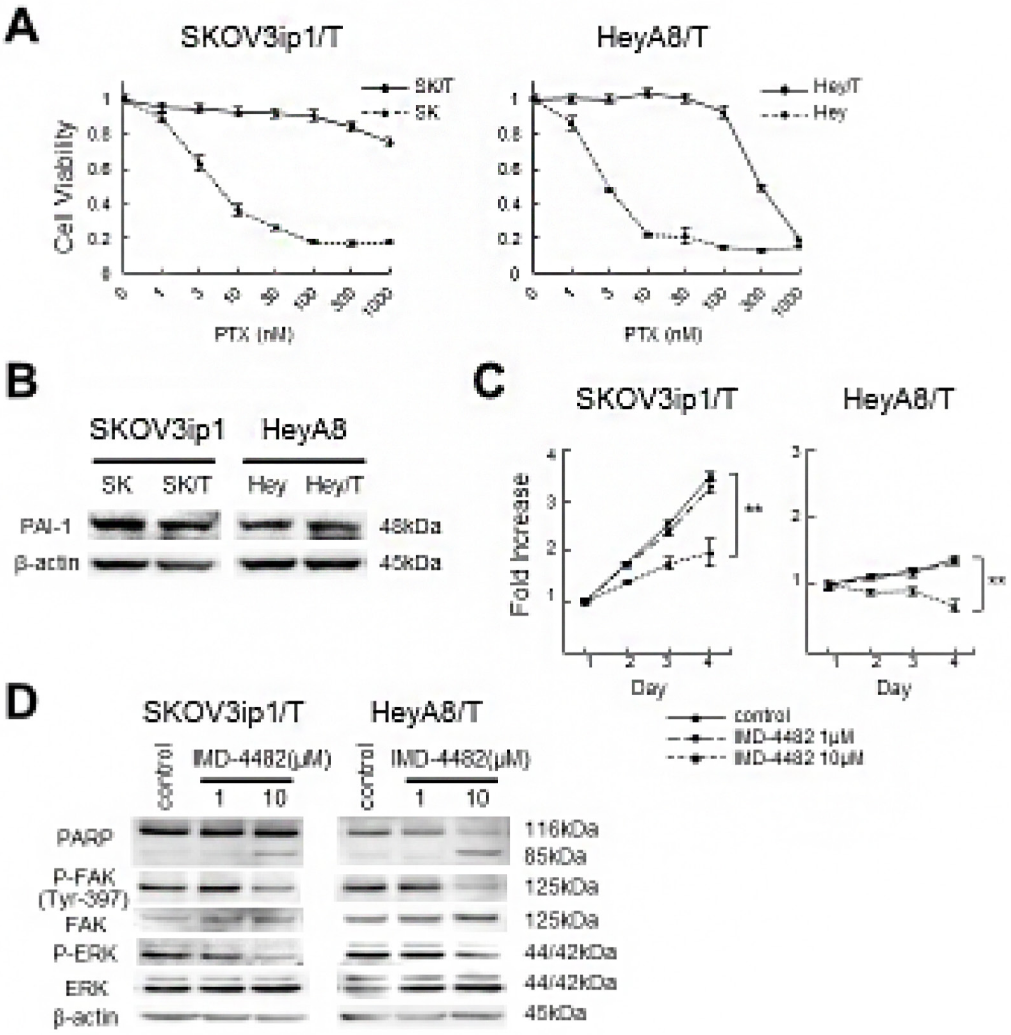

Supplementary Figure 3: IMD-4482 caused apoptosis in paclitaxel-resistant ovarian cancer cell lines. Establishment of paclitaxel resistant ovarian cancer cell lines **(A)**. Viability of SKOV3ip1, SKOV3ip1/T, HeyA8 and HeyA8/T cells exposed to PTX for 96 hr (SKOV3ip1) or 72 hr (HeyA8) was determined using MTS assay. Western blot **(B)**. PAI-1 expression in paclitaxel-resistant cells was analyzed. β-Actin was used as a loading control. Cell proliferation assay **(C)**. Cell proliferation was measured as previously described. **(D)** Paclitaxel-resistant cells were treated with or without IMD-4482 for 24 hours. Cell lysates were immunoblotted with an antibody against PARP, p-FAK (Tyr-397), FAK, p-ERK, ERK. β-Actin was used as a loading control.

Supplementary Table 1: Clinicopathological characteristics of patients with serous adenocarcinoma

Number of patients	54
Median age, y (range)	62 (29-88)
Median observation time of patients alive, mo (range)	25 (3-78)
FIGO stage, n (%)	
I	11 (20.3)
II	1 (1.9)
III	28 (51.9)
IV	14 (25.9)
Residual tumor (cm), n (%)	
≤1	36 (66.7)
>1	18 (33.3)
Prior chemotherapy, n (%)	
Yes	9 (16.7)
No	45 (83.3)
Adjuvant chemotherapy, n (%)	
TC / ddTC	46 (85.2)
PTX	1 (1.9)
None	7 (13.0)
Vital status, n (%)	
Living	36 (66.7)
Decreased	18 (33.3)
PAI-1 staining, n (%)	
Weak (1+)	9 (16.7)
Moderate (2+)	8 (14.8)
Strong (3+)	37 (68.5)

Supplementary Table 2: PAI-1 staining scores in all histological types

	PAI-1 staining			
**Histological type**	**n**	**Weak (%)**	**Moderate (%)**	**Strong (%)**
Serous papillary adenocarcinoma	54	9 (16.7)	7 (13.0)	38 (70.4)
Endometrioid adenocarcinoma	22	3 (13.6)	2 (9.1)	17 (77.3)
Clear cell carcinoma	37	10 (27.0)	5 (13.5)	22 (59.5)
Mucinous adenocarcinoma	20	2 (10.0)	4 (20.0)	14 (70.0)
Others	21	0 (0)	8 (38.1)	13 (61.9)
